# Cerebrospinal fluid liquid biopsy identifies primary CNS lymphoma in a patient with concurrent lung adenocarcinoma and indeterminate brain lesion

**DOI:** 10.1093/noajnl/vdag168

**Published:** 2026-06-29

**Authors:** Connor J Liu, Bryn M Burkholder, Aliyah Pabani, Shuodan Zhang, Matthias Holdhoff

**Affiliations:** Department of Neurosurgery, Johns Hopkins University School of Medicine, Baltimore, Maryland (C.J.L); Wilmer Eye Institute, Johns Hopkins University School of Medicine, Baltimore, Maryland (B.M.B.); Department of Oncology, Johns Hopkins University School of Medicine and The Sidney Kimmel Comprehensive Cancer Center at Johns Hopkins, Baltimore, Maryland (A.P., S.Z., M.H.); Department of Oncology, Johns Hopkins University School of Medicine and The Sidney Kimmel Comprehensive Cancer Center at Johns Hopkins, Baltimore, Maryland (A.P., S.Z., M.H.); Department of Oncology, Johns Hopkins University School of Medicine and The Sidney Kimmel Comprehensive Cancer Center at Johns Hopkins, Baltimore, Maryland (A.P., S.Z., M.H.)

**Cerebrospinal fluid (CSF) liquid biopsies are an emerging technology with the potential to transform diagnosis and clinical decision-making in neuro-oncology. We report a patient with newly identified enhancing intracranial lesions and a concurrent lung adenocarcinoma, in whom CSF next-generation sequencing identified lymphoma-associated alterations in MYD88, CD79B, as well as NOTCH2, raising suspicion for primary CNS lymphoma prior to tissue diagnosis. Subsequent stereotactic brain biopsy confirmed a diagnosis of diffuse large B-cell lymphoma (DLBCL). This case highlights the clinical utility of CSF liquid biopsy to achieve diagnostic clarity and potentially reduce reliance on invasive biopsies in carefully selected patients.** 

## Case Description

An 87-year-old woman with a history of hypertension, coronary artery disease, and osteoarthritis, initially presented to her optometrist with bilateral floaters and was diagnosed with uveitis. She was treated with topical and oral corticosteroids with partial improvement. Due to persistent symptoms, she was evaluated by an ophthalmologist and underwent a right eye vitrectomy with a biopsy that was submitted for cytopathology, flow cytometry, and HSV, VZV, and CMV viral PCR. Viral studies were negative and flow cytometry showed only scant viable lymphoid cells. Cytopathology revealed atypical lymphoid cells with scattered CD20 immunostaining, raising the concern for central nervous system primary CNS lymphoma (PCNSL) with ocular involvement. At that time, brain MRI demonstrated no intracranial abnormalities and PET imaging was negative for systemic disease.

**Figure 1.**
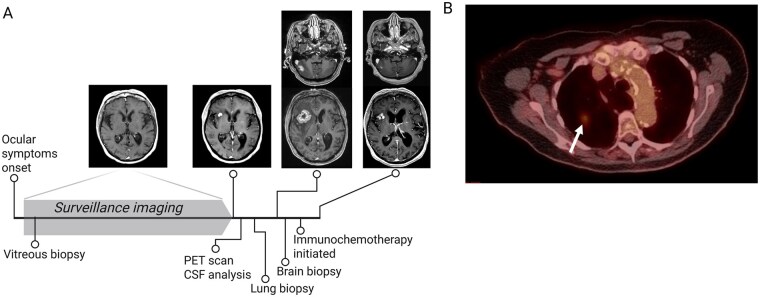
Clinical timeline, patient imaging, and CSF liquid biopsy results. (A) Clinical timeline from onset of this patient’s ocular symptoms (Day 0) to initiation of immunochemotherapy (Day 1149). Surveillance MRI (contrasted T1) imaging remained negative (left MRI), until 3 years after symptom onset (second MRI pictured) prompting diagnostic workup with CSF liquid biopsy. Rapid growth and additional enhancing lesions were demonstrated on short follow-up MRI (third MRI pictured). CSF cytology did not identify malignancy, so a brain biopsy was performed which confirmed CNS lymphoma. The patient was initiated on immunochemotherapy and a 1 month follow-up MRI demonstrated significant radiographic response (fourth MRI pictured). (B) Whole-body PET imaging identified a new hypermetabolic right upper lobe lung nodule (white arrow). A lung biopsy of this nodule confirmed moderately differentiated adenocarcinoma. Created using https://BioRender.com.

Over the next 2 years, she experienced intermittent ocular symptoms treated with oral and topical steroids. She was monitored with serial brain MRI imaging throughout this interval, which remained negative for intracranial disease (Figure 1A). Surveillance MRI obtained 3 years after symptom onset revealed a new 1.1 cm enhancing lesion in the right frontal lobe. This finding raised concern for PCNSL and initiated a broader work up, including a repeat PET scan to exclude systemic disease and CSF analysis for diagnostic confirmation. Her repeat PET scan showed a new hypermetabolic right upper lobe pulmonary nodule, and a lung biopsy was performed demonstrating moderately differentiated adenocarcinoma consistent with primary lung malignancy (Figure 1B). Given her advanced age, CSF liquid biopsy was performed to aid in diagnosis and potentially obviate the need for brain biopsy. Unfortunately, her CSF cytology and flow cytometry did not identify evidence of neoplastic involvement, including a monoclonal B-cell population. However, CSF NGS using the Belay Summit 2.0 assay, revealed multiple genomic alterations observed in diffuse large B-cell lymphomas (DLBCL), including MYD88 and CD79B copy number gains (Table 1).^1^^,^^2^ A short interval follow-up MRI demonstrated interval growth of her right frontal lesion as well as an additional new right cerebellar enhancing lesion.

**Table 1.** Relevant genetic alterations identified in CSF and lung biopsy

Alteration	Type	CSF VAF	Lung VAF	Present in CSF	Present in lung biopsy
ARID1B c.3346-2A>G	Splice-site mutation	92.80%	–	Y	N
MYD88 p.s243N	Single-nucleotide variant	61.70%	–	Y	N
NOTCH2 p. P2402fs	Frameshift deletion	41.80%	–	Y	N
PBRM1 p. S956fs	Frameshift insertion	0.40%	–	Y	N
CD79B	Copy number alteration	–	–	Y	N
HGF p. V631M	Single-nucleotide variant	–	47.94%	Y	Y

While clinical suspicion for a PCNSL remained high, the concurrent diagnosis of a primary lung neoplasm and the presence of new multicentric enhancing brain lesions introduced significant diagnostic uncertainty, with metastatic disease from the lung primary, lymphoma, and a primary glial neoplasm all remaining viable considerations. In hopes of clarifying the diagnostic conundrum, the patient underwent a right-sided stereotactic brain biopsy and pathology results confirming diffuse large B-cell lymphoma (DLBCL), ABC subtype, making the diagnosis of PCNSL. Given a good clinical status (Karnofsky Performance Status 90) and adequate renal and other organ function, despite her advanced age, the decision was made to treat the PCNSL with high-dose methotrexate-based induction therapy with curative intent. The patient initiated immunochemotherapy following the CALGB 50202 regimen with methotrexate, temozolomide, and rituximab (MTR) with the modified methotrexate dose to 3.5 g/m^2^ due to the patient’s age and frailty.^3^^,^^4^ After 2 cycles, follow-up MRI demonstrated significant size reduction in her enhancing right frontal and cerebellar enhancing lesions (Figure 1A). The patient tolerated treatment well, also showing signs of symptom improvement and subsequently completed definitive stereotactic radiosurgery with curative intent for her localized lung adenocarcinoma.

## Discussion

### Diagnostic Role of CSF Liquid Biopsy in PCNSL

This case illustrates the emerging clinical utility of CSF liquid biopsy in the diagnosis of indeterminate intracranial lesions for a patient with concurrent systemic malignancy. For this patient, conventional diagnostic approaches including MRI/PET imaging, vitreous biopsy, and CSF cytology were insufficient to diagnose PCNSL. Prior to CSF molecular profiling, the leading differential diagnoses included PCNSL, metastatic lung adenocarcinoma in the setting of the patient’s concurrent systemic malignancy, and less likely a primary glial neoplasm. Identification of lymphoma-associated alterations, including MYD88, substantially increased suspicion for PCNSL and reduced concern that the intracranial lesion represented metastatic lung cancer, particularly given the discordant molecular profiles between the CSF and lung biopsy specimens. Specifically, the assay identified MYD88 and NOTCH2 mutations, in addition to copy number gains involving CD79B, which, in the appropriate clinical context, strongly support the diagnosis of PCNSL. MYD88 mutations, particularly the p. L265P variant and, less commonly, the p. S243N, are highly recurrent in primary PCNSL, occurring in 50%-80% of cases (Table 1).^2^^,^^5^ In contrast, NOTCH2 alterations are less common and are not considered defining molecular features of primary PCNSL, although truncating NOTCH2 variants have been described across several B-cell lymphoma subtypes.^6^^,^^7^ Furthermore, compared to the recurrent CD79B p. Y196 hotspot mutation, CD79B copy number gains are not well described in the PCNSL literature. While tissue diagnosis remained necessary for definitive confirmation, the CSF results anticipated the final pathology and demonstrate how molecular profiling of CSF can provide clinically meaningful information earlier in the diagnostic workflow. In a frailer patient or in the setting of a less accessible lesion, similar CSF molecular findings may have supported empiric treatment without neurosurgical biopsy, as has been previously reported.^8^^,^^9^

### Assay Characteristics

In this case, CSF genomic profiling was performed using the Belay Summit 2.0 platform, a comprehensive NGS-based assay designed for molecular characterization of CNS malignancies. This assay utilizes NGS with high-depth sequencing (>10 000× coverage) on an Illumina platform, enabling detection of multiple variant classes, including single nucleotide variants and small insertions/deletions, copy number alterations, gene fusions, and broader genomic biomarkers such as tumor mutational burden and microsatellite instability for 520 genes. Notably, the assay requires relatively low input DNA, with reliable detection achieved using approximately 7.5 ng or more of CSF-derived total nucleic acid, making it well suited for low-volume CSF samples. Analytical validation studies have demonstrated high performance characteristics, including a sensitivity of approximately 96% for single nucleotide variants and insertions/deletions, with a limit of detection as low as 0.3% variant allele frequency, and overall clinical sensitivity and specificity of 96% and 98%, respectively.^1^ In this patient’s care, the turnaround time for CSF liquid biopsy results was 2 weeks, consistent with average estimates in clinical practice for tissue-based testing. While current costs remain higher than conventional cytology, they continue to decrease and are increasingly justified by the breadth of genomic information obtained. In addition, if one includes the cost of invasive brain biopsies, the relative cost of a liquid biopsy assay is a fraction of the expense associated with a surgical procedure.

### Clinical Implications and Future Directions

More broadly, liquid biopsy approaches based on analysis of cell-free DNA have rapidly advanced in neuro-oncology. CSF represents a particularly informative biofluid for central nervous system tumors, with a higher tumor DNA fraction compared to plasma and improved sensitivity for detecting tumor-specific alterations. As demonstrated in prior studies, these approaches can provide tumor classification, identify actionable alterations, and in some cases detect disease prior to radiographic progression.^10-15^ In addition, testing CSF as opposed to blood permitted reassurance that the genetic alterations identified were likely from the brain lesion as opposed to the concurrent lung cancer. Indeed, this paradigm has been described by others using CSF liquid biopsy with genotyping and ultrasensitive ctDNA profiling to accelerate diagnosis in PCNSL.^13-15^ However, this particular case highlights the clinical value of CSF liquid biopsy in resolving substantial diagnostic ambiguity in a patient with concurrent systemic malignancy. Apart from a single SNV overlap (HGF p. V631M), all genetic alterations were unique to either lung biopsy tissue (including a MET exon 14 skipping mutation) or CSF. These discordant genomic profiles between the CSF and the lung adenocarcinoma strongly supported the interpretation that the CSF alterations originated from the intracranial lesion rather than the systemic malignancy. Although NOTCH2 alterations have occasionally been reported in subsets of lung adenocarcinoma, particularly in tumors undergoing neuroendocrine transformation, MYD88 and CD79B variants are not recognized recurrent lung adenocarcinoma driver genes and are instead classically associated with B-cell lymphomas.^16^

Importantly, our case highlights the potential for CSF liquid biopsy to reduce reliance on invasive neurosurgical procedures in select clinical scenarios. To complete a conventional diagnostic workup, a total of 2 months passed from CSF liquid biopsy results to initiation of methotrexate-based chemotherapy, representing an opportunity for earlier intervention for a rapidly progressive pathology. While brain biopsy remains the gold standard for diagnosis, it carries inherent risks including hemorrhage, infection, neurological deficits, and anesthesia-related morbidity. Additionally, these considerations are compounded in more frail patients. In patients with characteristic molecular alterations identified in CSF and a consistent clinical and radiographic presentation, it is conceivable that CSF-based molecular diagnostics could obviate the need for surgical biopsy. Despite these promising findings, a negative CSF-NGS result for this patient would not have excluded PCNSL, particularly in the setting of prior corticosteroid exposure, limited CSF volume, or technical sampling constraints. Furthermore, molecular findings must additionally be interpreted within the broader clinical context, as false-positive results from clonal hematopoiesis or co-existing hematologic neoplasms remain theoretically possible.^17^ In the present case, these possibilities were considered less likely given the concordant radiographic presentation, the presence of multiple lymphoma-associated alterations, and the discordant genomic profile of the patient’s lung adenocarcinoma. Contemporary World Health Organization (WHO) and National Comprehensive Cancer Network (NCCN) guidelines increasingly emphasize molecular characterization and CSF-tDNA testing in the diagnostic evaluation of CNS tumors, including PCNSL.^18^ Beyond its diagnostic role, CSF liquid biopsy may also enable longitudinal disease monitoring. Serial assessment of tumor-derived DNA in CSF has been shown to track treatment response, detect minimal residual disease, and identify recurrence prior to imaging changes.^19^^,^^20^

To date, the patient has completed 4 cycles of methotrexate, rituximab, and temozolomide (MTR). Follow-up MRI obtained approximately 1 month after treatment initiation demonstrated a significant reduction in the size of the enhancing right frontal and right cerebellar lesions, consistent with a partial radiographic response (Figure 1). At last follow-up, the patient was clinically well with preserved functional status and activities of daily living and with no evidence of neurotoxicity or major treatment-related complications. Thus, for neuro-oncology patients in whom tissue specimens are too high risk to obtain, liquid biopsy may unlock targeted therapy trials or treatments for which they otherwise would not have been eligible for without a tissue diagnosis. As CSF liquid biopsy approaches gain additional prospective validation, they represent an advancing landscape of enhanced clinical and therapeutic decision-making in neuro-oncology.

## Data Availability

The data underlying this article are contained within the article. No supplementary material accompanies this manuscript.
